# Multisensory Facial Stimulation Implicitly Improves Evaluations of the Goodness of Attractive Others

**DOI:** 10.3389/fpsyg.2019.01239

**Published:** 2019-05-29

**Authors:** Ji Woon Jeong, Eunhee Chang, Hyun Taek Kim

**Affiliations:** Department of Psychology, Korea University, Seoul, South Korea

**Keywords:** visuotactile multisensory integration, enfacement effect, implicit evaluation, single category implicit association test, face attractiveness

## Abstract

It has been well demonstrated that shared multisensory experiences between the self and others can influence the social perception of out-group members. Previous research has shown that the illusion of ownership over a dark-skinned rubber hand or full virtual body generated less negative implicit bias against people with dark skin. However, less is known about how perceived attractiveness difference between self and other affects social perception toward those others after shared multisensory experience. The present study assessed whether shared multisensory experience between the self and attractive others would affect the implicit evaluation of goodness of others. Seventy-three women participated in the study. After the visuotactile multisensory stimulation procedure, participants were administered the Single Category Implicit Association Test (SC-IAT), which presents two attributes (good and bad) and one concept (other). Results showed that the more attractive the faces are, the more positive their implicit evaluation becomes after the synchronous tactile stimulation. This result suggests that shared multisensory experience makes people feel more positive toward others who have positive attribute. This finding suggests that self-other blurring in social contexts might be a compelling factor in evaluating other people positively.

## Introduction

Previous studies on the enfacement effect—a type of self-other face perception modification—have shown that interactions between various types of bottom-up sensory information (i.e., multisensory integration) can play an important role in modulating the physical boundary between the self and others (Tsakiris, 2008; Paladino et al., 2010; Sforza et al., 2010; Tajadura-Jimenez et al., 2012). Specifically, the enfacement effect is a result of the interaction between vision and touch; watching a face on a monitor being touched in synchrony with one’s own face could cause the subject to feel as though the face in the monitor is his or her own.

Tsakiris (2008) reported on how multisensory integration can change an individual’s own facial representations. In that study, subjects who received synchronous visuotactile stimulation presented an increased tendency to recognize morphed faces as their own. Similar effects were reported in the study by Sforza et al. (2010). In their experiment, visuotactile stimulation was delivered to the participant’s face as well as to a partner’s face. The enfacement effect was seen after synchronous stimulation rather than after asynchronous stimulation and the effect correlated with the participant’s empathic traits and with the physical attractiveness of their partner. Later, Tajadura-Jimenez et al. (2012) provided objective psychophysiological evidence of changes in the perception of others’ faces following synchronous visuotactile stimulation. In their study, subjects’ autonomic responses, such as heart rate deceleration and electrodermal activity, increased when a threatening object approached the other person’s face subsequent to synchronous multisensory stimulation.

Furthermore, studies have investigated how illusory ownership over a hand or a full body induced by multisensory integration can change participants’ implicit racial bias, indicating that such an effect extends beyond bodily perception to social perception (Farmer et al., 2012; Maister et al., 2013; Peck et al., 2013). For instance, the strength of the body ownership that was experienced predicted the participants’ implicit racial bias indicating that those who experienced a stronger rubber hand illusion showed lower racial bias (Farmer et al., 2012; Maister et al., 2013). Similarly, the illusion of ownership over a full virtual body after visuomotor synchrony reduced implicit racial bias toward dark-skinned people (Peck et al., 2013). These results revealed that even strong social prejudice such as racial bias could be modulated by using experimental manipulation to increase self-other overlap.

Although the studies mentioned above provided evidence of social perception changes induced by illusory ownership over a hand or a full body, only a few studies directly investigated the social effect of illusory facial ownership. For instance, Paladino et al. (2010) demonstrated that participants exposed to synchronous visuotactile stimulation with other’s face showed a high level of self-other merging, as indicated by the participant’s perception of face resemblance as well as the participant’s judgment of the inner state of the other and by the feeling of a greater degree of closeness toward the other. Moreover, these participants showed increased conformity behavior compared to participants exposed to asynchronous stimulation. In the study by Sforza et al. (2010), modification of self-face recognition was induced by synchronous multisensory stimulation and this enfacement effect was correlated positively with the physical attractiveness that the participants attributed to their partner.

Meanwhile, it is well known that people usually categorize others as either similar to oneself or dissimilar based on body-related visual information (Leonardelli and Toh, 2015). Demographic dissimilarity such as race or gender would be one of the most salient and obvious body-related visual cues to categorize themselves and others in social contexts. Although, a basic form of categorization of others is based on demographic characteristics, categorization can also be based on a variety of features that are behavioral (e.g., participation in a sports club or playing piano), attitudinal or ideological (gay liberation or conservatism), or dispositional (e.g., passionate or optimistic), as well as physical (e.g., makeup or hair styles) (Leonardelli and Toh, 2015) and would influence the development of social bias. Among them, physical attractiveness would be one of the most apparent information in order to perceive difference between self and other and appears to be a method of participating in social cognition processes, i.e., stereotyping such as “what is beautiful is good”(Dion et al., 1972; Landy and Sigall, 1974; Rhodes, 2006). So far, however, less is known about how perceived attractiveness difference between the participant’s own face and other’s face affects social perception toward those others after multisensory integration procedure.

In the studies by Farmer et al. (2012) and by Maister et al. (2013), the authors manipulated the skin colors of the rubber hand (i.e., black and white) to examine whether white participants experienced a sense of body ownership for a body part from a same or different racial group and found that the effect was specific to implicit attitudes toward an out-group skin color. Similarly, in this study, the authors needed to manipulate the difference in attractiveness levels to distinguish between oneself and other. Skin color is obvious physical information that distinguishes me from other whereas perceived attractiveness difference between me and other are relatively subjective and depends on the bias of the observer. Previous studies have suggested that self-rated attractiveness might contain systematic variance related to people’s tendencies to view their appearance overly negatively or positively (Weeden and Sabini, 2007). It means that if I perceive other’s face as attractive and my face as attractive as well, there would be no perceived attractiveness difference between other and me. Therefore, manipulation of the attractiveness level based on both judgments for their own face as well as other’s face is necessary to make perceived attractiveness different.

We predicted that synchronous tactile stimulation while watching a perceived attractive face being touched would positively affect subjects’ evaluations of others. Maister et al. (2015) provided an explanation how multisensory integration could change participants’ social perception of out-group members. Maister et al. (2015) suggested that the perceived physical similarity between oneself and out-group members might increase at the beginning (self-association in the bodily domain), which in turn leads to a generalization of positive self-like associations to the out-group at the conceptual level. If this hypothesis is true, then the illusory body ownership should positively affect the evaluation of others.

In sum, this study aimed to expand previous observations that have indicated that shared multisensory experience has positively modulated subjects’ social perceptions. This study assessed participants’ evaluation toward an individual rather than the social bias regarding the out-group as a whole. For this purpose, we used the Single Category Implicit Association Test (SC-IAT) (Karpinski and Steinman, 2006), which presents two attributes (good and bad) and one concept (other). The SC-IAT was administered immediately after synchronous or asynchronous visuotactile stimulations to measure the implicit evaluation of others. Only young female subjects participated in this study, as it has been reported that women tend to be more strongly affected by the physical attractiveness of others relative to men (Brown and Gallagher, 1992; Gutierres et al., 1999; Wade and Cooper, 1999).

## Materials and Methods

### Subjects

Seventy-three healthy women (mean age = 21.2 years, SD = 2.3 years) participated in the experiment. All subjects had normal or contact lens corrected-to-normal vision (i.e., none wore glasses) and reported normal tactile perception.

### Ethics Statement

All subjects provided written informed consent before participating in the experimental procedures, which were approved by the ethics committee of Korea University (1040548-KU-IRB-13-65-A-2). The individuals in this manuscript have given written informed consent to publish these case details.

### Materials

#### Experimental Stimuli

A photograph of each subject’s face was taken before the experiment using a digital camera. The experimenter instructed subjects to pull back their hair if it fell onto their faces and to make neutral facial expressions in the photograph. These images were cropped and edited using Adobe Photoshop CS3, Extended Version 10.0 (Adobe Systems, USA), to remove nonfacial attributes (e.g., background, hair, ears) and create a uniform black background.

A computerized morphing program (Abrosoft FantaMorph version 5.4.1; Abrosoft, USA) was used to produce an averagely attractive (AA) face and 12 highly attractive (HA) faces. To create the 12 HA faces, faces of attractive Korean actresses with neutral facial expressions were collected from web pages, and those with the eyes and face oriented forward were selected. The facial images of attractive actresses were edited in the same manner as the self-face. Twelve HA faces were produced by morphing the faces of two attractive actresses. Among them, the most attractive face chosen by each subject was used in the experiment. The AA face was produced by morphing two anonymous faces from the Korea University Facial Expression Collection (Kim et al., 2011).

Subjects rated their own facial attractiveness, an AA face, and 12 HA faces on a 7-point Likert scale immediately before the experiment. The ratings of facial attractiveness were significantly different, *χ*^2^(2) = 124.32, *p* < 0.001. HA faces were rated as more attractive, *M* = 6.22, SD = 0.95 relative to AA faces, *M* = 3.99, SD = 0.86; *z* = −7.23, *p* < 0.001. Furthermore, subjects perceived both the HA faces and the AA face as more attractive than their own faces, *M* = 2.92, SD = 1.21; HA: *z* = −7.83, *p* < 0.001; AA: *z* = −6.14, *p* < 0.001.

The AA and HA faces were used to produce 2-min movies showing the face being touched on the right cheek with a small paintbrush that moved in a constant rhythm diagonally to the right and upward. The movies were produced using Adobe Premiere Pro version 7.0 (Adobe Systems, USA).

#### Single Category Implicit Association Test

In the SC-IAT, five idiographic items related to the other (i.e., name, birth year, birth date, last four digits of mobile phone number, and academic major) were used as concept categories. The lists of good and bad attributes were selected from the item pool used by Greenwald and Farnham (2000). Fifty-two good and bad attributes were rated on a scale from 1 (very negative) to 7 (very positive) by 10 subjects (mean age: 27.6 ± 4.3 years) who did not participate in the experiment. The 10 most positive attributes (e.g., “loved” and “peace”) and 10 most negative attributes (e.g., “hated” and “torture”) were used in the experiment. There was a significant difference in ratings between good and bad attributes (good attributes: *M* = 6.5, SD = 0.2; bad attributes: *M* = 1.3, SD = 0.2; *t*(9) = 39.94, *p* < 0.001; paired *t*-test).

#### Face Illusion Questionnaire

The Face Illusion Questionnaire (FIQ) was adapted from by Longo et al. (2008) to measure specific perceptual experience after synchronous and asynchronous multisensory stimulation. The questionnaire consists of 10 questions assessing sense of ownership (i.e., the feeling of becoming the person in the monitor, Q1–Q5); whether subjects confused the locations of the touches that were seen and those that were felt (i.e., confusion regarding the location of the stimulation, Q6–Q8); and agency of control (i.e., the sensation of controlling the face in the monitor, Q9–Q10). Each question is rated on a 7-point scale from 1 (not at all) to 7 (extremely). The 10 questions are as follows:

Q1. It seemed like I was looking directly at my own face, rather than at a face in the monitor.
Q2. It seemed like the face began to resemble my real face.
Q3. It seemed like the face in the monitor belonged to me.
Q4. It seemed like the face in the monitor was my face.
Q5. It seemed like the face in the monitor was part of my body.
Q6. It seemed like my face was in the location where the face in the monitor was.
Q7. It seemed like the face in the monitor was in the location where my face was.
Q8. It seemed like the touch I felt was caused by the paintbrush touching the face in the monitor.
Q9. It seemed like I could have moved the face in the monitor if I had wanted.
Q10. It seemed like I was in control of the face in the monitor.

### Procedures

**Figure 1:**
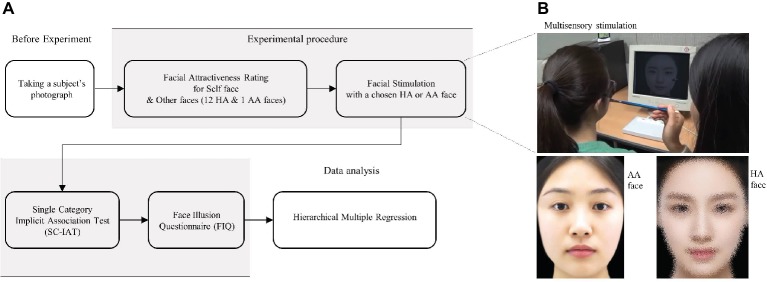
**(A)** Schematic time line of experiment. **(B)** Visuotactile multisensory stimulation and an example of averagely attractive (AA) and highly attractive (HA) faces. Subjects’ faces were touched with a paintbrush while they viewed AA or HA faces being touched in or out of synchrony with their own faces (upper). The HA face image was pixelated to avoid copyright infringement (lower right).

The schematic representation of the experimental procedure is depicted in Figure 1. One to 3 days after their photographs were taken, subjects participated in the experiment. They were randomly assigned to one of four conditions (i.e., synchronous/AA (*n* = 19), synchronous/HA (*n* = 18), asynchronous/AA (*n* = 18), and asynchronous/HA (*n* = 18) conditions).

During stimulation, the movie was presented in full screen mode on a 17-inch flat-screen CRT monitor (Samsung, Korea) positioned at a distance of 50 cm from the subjects. Subjects were instructed to focus on the movie on the monitor screen as soon as possible after it began and not move. They were also instructed to maintain a neutral facial expression. Subjects wore glasses that had been modified by removing the lenses and attaching blinkers to the frames so that they could remain focused on the faces presented on the computer monitor.

The trained experimenter touched subjects’ right cheeks with a paintbrush (1.0 cm × 1.2 cm) while they were watching the movie showing the face being touched on the right cheek with an identical paintbrush. In the synchronous condition, while the subjects were looking at a face, they were simultaneously touched in the same place that the other face was being touched. In the asynchronous condition, on the other hand, the temporal and directional congruency between the stimulus felt on one’s own face and the identical stimulus observed on the other person’s face on the monitor was disrupted. The stimulation covered a distance of approximately 2 cm on the face.

Subjects completed the SC-IAT for the other after stimulation (Table 1). The SC-IAT consisted of two stages and all subjects completed the task in the same order (Karpinski and Steinman, 2006). Each stage consisted of a practice block (24 trials) followed by a test block (72 trials). Instructions and key assignments preceded each block.

**Table 1:** Arrangement of the SC-IAT blocks.

Block	No. of trials	Function	Items (left key)	Items (right key)
1	24	Practice	Good/other	Bad
2	72	Test	Good/other	Bad
3	24	Practice	Good	Bad/other
4	72	Test	Good	Bad/other

In the SC-IAT, all stimuli appeared in the center of the computer screen, and subjects were asked to classify these stimuli as quickly and accurately as possible by pressing one of two response keys. In each trial, the stimulus was presented until the subject responded or until 1,500 ms had passed; if subjects failed to respond within 1,500 ms, a reminder—“Please respond more quickly”—appeared for 500 ms. If a response was incorrect, subjects were provided with feedback *via* a red “X” in the center of the screen for 150 ms; however, they were not required to correct incorrect responses. If a response was correct, a green “O” was presented in the center of the screen for 150 ms.

When the experiment concluded, subjects were asked to complete the FIQ. They were then debriefed about the study.

### Data Analysis

SC-IAT scores were computed by using the D-score algorithm described by Karpinski and Steinman (2006), which is modeled on the D-score algorithm used for IAT data. The 24 practice trials in each stage (i.e., blocks 1 and 3) were discarded. Trials with latencies of less than 350 ms and nonresponse trials were excluded from data analysis. Latencies for error trials were replaced with the mean RT for the block in which the error occurred plus a penalty of 400 ms. The average RT of block 2 was subtracted from the average RT of block 4. The difference score was divided by the standard deviation of all corrected RTs within blocks 2 and 4. Higher D-scores for the other evaluation indicated more positive attitudes toward the other.

In order to calculate the perceived attractiveness difference, the scores of self-face attractiveness ratings were subtracted from the scores of attractiveness ratings of HA or AA faces. The scores of perceived attractiveness difference ranged from −1 to +6. All statistical analyses were performed based on these scores.

The mean ratings of facial attractiveness for each face stimulus were compared by using Friedman’s ANOVA and Wilcoxon signed-rank tests. Regression was chosen as appropriate method for the statistical analysis because our experiments involved both categorical (stimulation mode) and continuous (perceived attractiveness difference and ownership) variables. Because effect of ownership on implicit attitude change was shown to be highly significant in the previous research (Maister et al., 2013), ownership was considered as a variable in the analysis. The two-step hierarchical linear regressions were conducted on mean scores of subscale of the FIQ as well as D-scores from the SC-IAT for the others. Two continuous variables that were used to calculate interaction terms were mean-centered before being added into the regressions to minimize multicollinearity. All statistical tests were two-tailed using a significance level of *p* < 0.05. Furthermore, *post hoc* power analysis was conducted with G*power 3.1 (Faul et al., 2009), with an alpha level of 0.05 and a total sample size of 73 participants (reported for all significant findings in the results section).

## Results

### Facial Illusions

Mean scores of subscales of the FIQ, i.e., ownership (Q1–Q5), location (Q6–Q8), and agency (Q9–Q10) were used as dependent variables in linear regressions with stimulation mode and perceived attractiveness difference as predictor variable in the first step, and as a two-way interaction term entered in the second step. For the ownership score, the overall model fit was significant at the first step, *F* (2,72) = 10.514, *p* < 0.001, $\eta_{p}^{2}$ = 0.231; power = 0.99. Stimulation mode significantly predicted the experience of ownership, β = 0.445, *p* < 0.001, indicating exposure to synchronous stimulation led to a greater illusion of ownership to the face in the monitor. Adding the interaction term to the model in the second step of the regression did not significantly improve the model fit, Δ*R*^2^ = 0.000, *F* (3,72) = 6.918, *p* < 0.001, $\eta_{p}^{2}$ = 0.231; power = 0.98. For the location score, the overall model fit was significant at the first step, *F* (2,72) = 10.106, *p* < 0.001, $\eta_{p}^{2}$ = 0.224; power = 0.99, but not at the second step, Δ*R*^2^ = 0.000, *F* (3,72) = 6.648, *p =* 0.001, $\eta_{p}^{2}$ = 0.224; power = 0.97. Stimulation mode significantly predicted the illusion of location, β = 0.463, *p* < 0.001. For the agency score, the overall model fit was also significant at the first step, *F* (2,72) = 4.140, *p =* 0.020; power = 0.73, $\eta_{p}^{2}$ = 0.106, but not at the second step, Δ*R*^2^ = 0.000, *F* (3,72) = 2.720, *p =* 0.051, $\eta_{p}^{2}$ = 0.106. Stimulation mode significantly predicted the illusion of location, β = 0.266, *p =* 0.021.

### Single Category Implicit Association Test

D-scores from SC-IAT for other were analyzed in a linear regression with stimulation mode, perceived attractiveness difference, and ownership as predictor variables in the first step and two-way interaction terms entered in the second step. As presented in Table 2, the overall model fit was significant at the first step, *F* (3,72) = 2.831, *p =* 0.045, $\eta_{p}^{2}$ = 0.110; power = 0.69. Stimulation mode significantly predicted D-score, β = 0.302, *p =* 0.021. Adding the interaction term to the model in the second step of regression significantly improved the model fit, Δ*R*^2^ = 0.071, *F* (6,72) = 2.428, *p =* 0.035, $\eta_{p}^{2}$ = 0.181; power = 0.83. The interaction of stimulation mode and perceived attractiveness difference significantly predicted D-score, β = 0.425, *p =* 0.024. Simple linear regressions on D-score, with perceived attractiveness difference entered as a predictor variable, were carried out for the synchronous and the asynchronous conditions.

**Table 2:** Summary of two-step hierarchical regression analysis for variables predicting D-score.

Variable	*β*	*p*
**Step1**		
Stimulation mode	0.302	0.021
Perceived attractiveness	0.106	0.911
Ownership	0.027	0.835
**Step2**		
Stimulation mode × perceived attractiveness	0.425	0.024
Stimulation mode × ownership	0.075	0.687
Perceived attractiveness × ownership	−0.084	0.522

Note. $R_{\mathrm{adjusted}}^{2}$ = 0.071 for step 1 (*p =* 0.045); Δ*R*^2^ = 0.071 for step 2 (*p =* 0.035).

**Figure 2:**
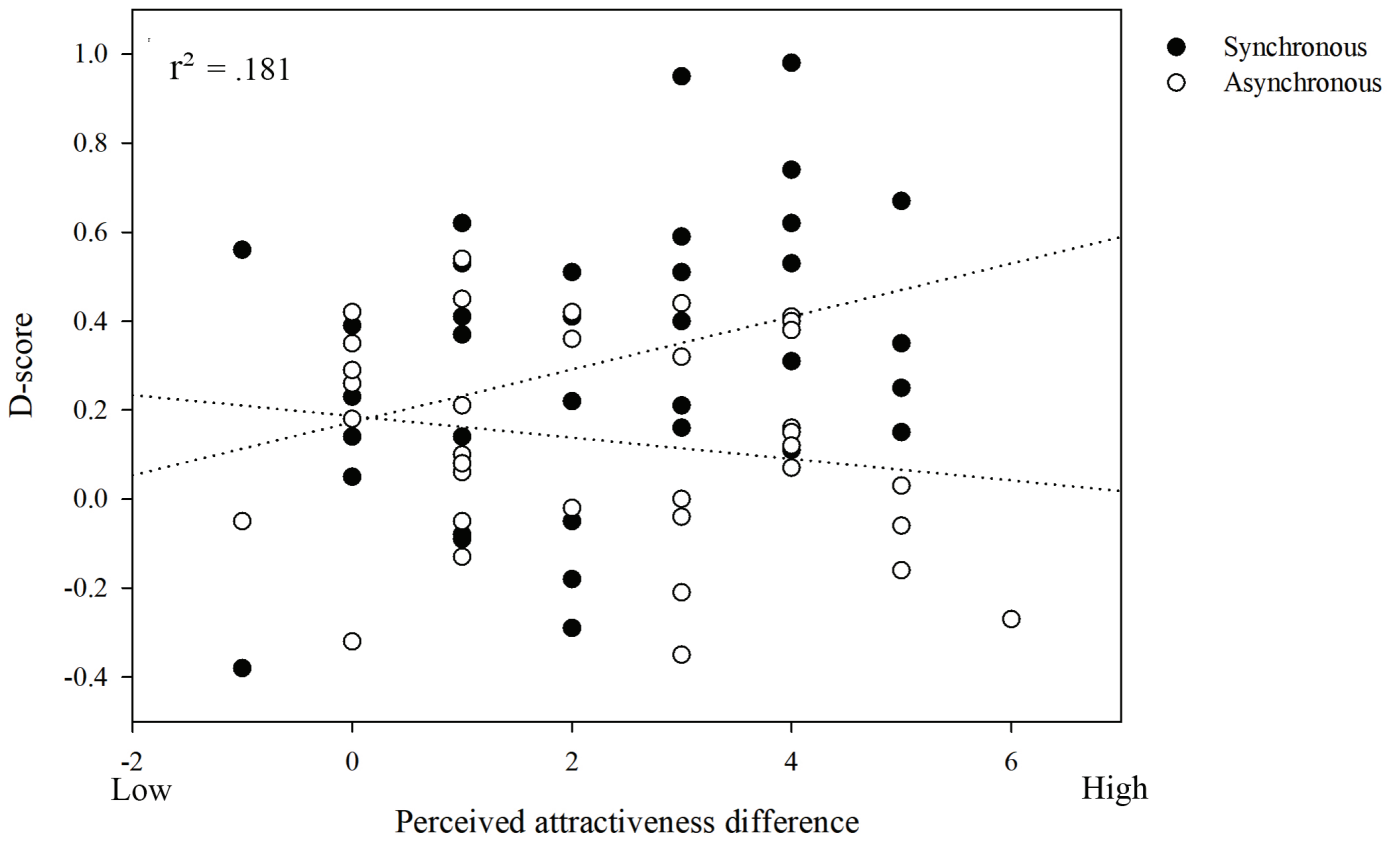
Perceived attractiveness difference significantly predicted D-score in the synchronous condition. The more attractive the faces are, the more positive their implicit evaluation becomes after the synchronous tactile stimulation. However, in asynchronous stimulation condition, perceived attractiveness difference was not a significant predictor of D-score.

Perceived attractiveness difference significantly predicted D-score in the synchronous condition, β = 0.333, *p =* 0.044. After synchronous stimulation, the more attractive the faces are, the more positive their implicit evaluation becomes. However, in asynchronous stimulation condition, perceived attractiveness difference was not a significant predictor of D-score, β = −0.183, *p =* 0.285. These findings are presented in Figure 2.

## Discussion

The present study investigated whether shared multisensory experience could affect the implicit evaluation of goodness of others who are more attractive than themselves. The first aim of this study was to investigate if synchronous multisensory stimulation could affect participant’s social perception to others who are different from oneself in terms of attractiveness level. The second aim was to investigate whether the self-other merging could affect the evaluation of others in a positive direction in the case of an attractive face. Finally, the authors would like to show that multisensory integration could also manipulate the implicit evaluation toward an individual, not just toward an out-group such as a racial out-group. The results show that the more attractive the faces are, the more positive their implicit evaluation becomes after the synchronous tactile stimulation. The asynchronous tactile stimulation did not affect evaluations of the other. Taken together, shared multisensory experiences between the self and attractive others affect evaluations of that attractive other (i.e., make them appear to be “good persons”).

While most previous studies showed a decrease in negative implicit attitudes such as implicit racial bias toward dark-skinned people (Maister et al., 2013; Peck et al., 2013), this study found a significant increase in the implicit evaluation of others who are different from oneself in terms of attractiveness levels. The authors manipulated facial attractiveness, because it may be considered that attractiveness, as the positive attribute of the face, is one of the few variables to affect implicit social attitude. Consistent with most previous studies, our results also demonstrated that illusory ownership positively modulated subjects’ social perception (Farmer et al., 2012; Maister et al., 2013; Peck et al., 2013).

Shared multisensory experiences affected one’s evaluations of the goodness of others the more the participants judged others’ faces as more attractive than themselves. In other words, the goodness of others may not be modulated by shared multisensory experience if the other had a similar level of attractiveness. This result is consistent with a previous study in which light-skinned Caucasian subjects participated and illusory ownership was induced over either a dark- or light-skinned rubber hand (Maister et al., 2013). In this study, the more intense the participants’ ownership over the dark-skinned rubber hand, the more positive the implicit attitude becomes, but this effect was specific to the out-group (i.e., dark-skinned people). These results confirmed Maister’s perspective (Maister et al., 2015), which claimed that changing social perception about an out-group is preceded by an increased physical similarity due to shared multisensory experiences. That is, if there is no physical difference between oneself and others from the beginning, then social perception will not be affected.

While it has been well demonstrated that the experience of ownership over an out-group body results in changes in the implicit bias against that out-group (Maister et al., 2013; Peck et al., 2013), it was not clear whether it also could manipulate the implicit evaluation of an individual as well. Although Paladino et al. (2010) showed shared multisensory experience can change the social perception for individuals, they explicitly measured self-other merging such as perceptions of resemblance, judgments of the other’s inner state, closeness felt toward the other. This study demonstrated the experimental evidence that shared multisensory experience can affect implicit social perception to others. Previous studies using the Race Implicit Association Test have shown that shared multisensory experience affected subjects’ implicit attitude toward the out-group itself rather than each individual in the out-group. In fact, even if I am a white person with racial prejudice, I can have a positive attitude toward someone who is black as well as a negative attitude toward someone who is white. Therefore, this study assessed participant’s evaluation toward an individual rather than the social bias regarding the out-group as a whole.

We did not find the correlation between ownership and implicit evaluation of others (Maister et al., 2013). In the studies using rubber hand in the multisensory stimulation, the overall strength of experienced hand ownership predicted the participant’s post-illusion implicit racial bias, with those who experienced a stronger illusory ownership showing a lower bias. Because sense of identity linked to the face is much more hardwired and stable than that linked to hand or the full body (Sforza et al., 2010), the results from the studies using hand and face could not be directly compared to the present study. As they suggested, there is the possibility that the component of the illusion related to facial ownership might be less reliably measured at the subjective, phenomenological level. They suggested changes of sense of facial identity after the multisensory stimulation can be induced more easily when tested by means of self-other morph rating task with respect to when tested by means of a task based on the report of subjective phenomenological experiences. Further investigations will be needed to directly demonstrate the relationship between the results of a variety of tasks and D-scores.

The limitation of our study can be inferred directly from the fact that the SC-IAT was performed only after the visuotactile stimulation was administered, but not before. It generated a possible criticism, in which the observed differences were not uniquely attributable to experimental manipulations but owing to baseline differences. According to the authors’ unpublished data, there was no group difference based on the attractiveness difference in the baseline. In the double-category IAT, which contrasts self and other target categories, D-scores were −0.59 and −0.56 for average attractive face and highly attractive faces, respectively (see Supplementary Materials). In the future, it will be necessary to present data proving that there is no observable difference in the baseline.

In summary, shared multisensory experiences between the self and attractive others would affect the implicit evaluation of the goodness of those others. This study first reveals that shared multisensory experience can implicitly affect the social perception toward an individual. This study also suggests the possibility that shared multisensory experience usually makes people feel more positive toward others who have positive attributes. This finding confirms previous research indicating that shared multisensory experience affects subjects’ social perceptions of others. Self-other blurring in social contexts might be a compelling factor in evaluating other people positively.

## Ethics Statement

This study was carried out in accordance with the recommendations of 1040548-KU-IRB-13-65-A-2 with written informed consent from all subjects. All subjects gave written informed consent in accordance with the Declaration of Helsinki. The protocol was approved by the institutional review board of Korea University.

## Author Contributions

JJ and HK conceived the study and participated in its design. JJ conducted the experiment. JJ, EC, and HK drafted the article and revised it critically for important intellectual content. All authors read and approved the final manuscript.

### Conflict of Interest Statement

The authors declare that the research was conducted in the absence of any commercial or financial relationships that could be construed as a potential conflict of interest.
