# Supplementary material for: Multisensory Facial Stimulation Implicitly Improves Evaluations of the Goodness of Attractive Others
Source: Front Psychol. 2019 May 29;10:1239. doi: 10.3389/fpsyg.2019.01239 (PMC6563892; doi:10.3389/fpsyg.2019.01239)
Supplement: Supplementary file 1 [file Table_1.docx]

Supplementary Material

# Supplementary Table

Baseline scores in double-category IAT (DC-IAT)

|  | | Baseline scores in DC-IAT | | | | | |
| --- | --- | --- | --- | --- | --- | --- | --- |
|  | Averagely attractive face | | | |  | Highly Attractive face | |
|  | Self | | Other |  | | Self | Other |
| Good evaluation | 573.9 ± 63.6 | | 714.5 ± 106.8 |  | | 574.3 ± 81.1 | 720.7 ± 109.7 |
| Bad evaluation | 677.6 ± 136.9 | | 584.3 ± 72.8 |  | | 700.6 ± 138.4 | 593.2 ± 99.0 |
| D-Score | 0.5 ± 0.3 | | -0.6 ± 0.5 |  | | 0.6 ± 0.4 | -0.6± 0.3 |
